# Combined statin and angiotensin-converting enzyme (ACE) inhibitor treatment increases the lifespan of long-lived F1 male mice

**DOI:** 10.1007/s11357-016-9948-4

**Published:** 2016-09-02

**Authors:** Stephen R. Spindler, Patricia L. Mote, James M. Flegal

**Affiliations:** 1 Department of Biochemistry, University of California at Riverside, Riverside, CA 92521 USA; 2 Department of Statistics, University of California at Riverside, Riverside, CA 92521 USA

**Keywords:** Statins, ACE inhibitors, Angiotensin II receptor antagonists, Life span, Longevity

## Abstract

Statins, such as simvastatin, and ACE inhibitors (ACEis), such as ramipril, are standard therapies for the prevention and treatment of cardiovascular diseases. These types of drugs are commonly administered together. More recently, angiotensin II type 1 receptor (AT1R) antagonists, such as candesartan cilexetil (candesartan), have been used in the place of, or in combination with, ACEis. Here, we investigated the effects of simvastatin and ramipril single and combination therapy, and candesartan treatment on the lifespan of isocalorically fed, long-lived, B6C3F1 mice. Males were used for their relative endocrine simplicity and to minimize animal usage. The drugs were administered daily in food. The simvastatin and ramipril combination therapy significantly increased the mean and median lifespan by 9 %. In contrast, simvastatin, ramipril, or candesartan monotherapy was ineffective. All groups consumed the same number of calories. Simvastatin, alone or administered with ramipril, decreased body weight without changing caloric consumption, suggesting it may alter energy utilization in mice. Combination therapy elevated serum triglyceride and glucose levels, consistent with altered energy homeostasis. Few significant or consistent differences were found in mortality-associated pathologies among the groups. Simvastatin treatment did not reduce normal serum cholesterol or lipid levels in these mice, suggesting that the longevity effects may stem from the *pleiotropic*, non-cholesterol-related, effects of statins. Together, the results suggest that statins and ACEis together may enhance mouse longevity. Statins and ACE inhibitors are generally well-tolerated, and in combination, they have been shown to increase the lifespan of normotensive, normocholesterolemic humans.

## Introduction

Cardiovascular disease is the leading cause of morbidity and mortality worldwide (World Health Organization 2004). Statins [3-hydroxy-3-methylglutaryl coenzyme A (HMG-CoA) reductase inhibitors], such as simvastatin, reduce the biosynthesis of the isoprenoids used for cholesterol biosynthesis (Spindler et al. 2012a; Edwards and Ericsson 1999). Statins have been shown by meta-analyses to reduce cardiovascular death by 20–30 % (Taylor et al. 2011). These health benefits appear to stem from both reduced serum cholesterol levels (Ludman et al. 2009) and reduced protein isoprenylation [e.g., (Spindler et al. 2012a)]. The *pleiotropic effects* of reduced isoprenoid biosynthesis are independent of their effects on cholesterol levels (Spindler et al. 2012a; Ludman et al. 2009). For example, simvastatin’s non-cholesterol-related, pleotropic effects increase the lifespan and health span of *Drosophila* by decreasing protein isoprenylation (Spindler et al. 2012a).

ACEis, such as ramipril, are used as antihypertensives (Crowley et al. 2012). They reduce the biosynthesis of angiotensin and thereby reduce the activity of the AT receptors, most importantly, AT1R [reviewed in (te Riet et al. 2015)]. In the vessel wall, AT1R activation induces vasoconstriction, endothelial dysfunction, inflammation, growth, and remodeling [reviewed in (te Riet et al. 2015)]. Reduced AT1R signaling reduces blood pressure and the incidence of stroke, diabetic kidney disease, congestive heart failure, diabetes mellitus, and atrial fibrillation [reviewed in (Strauss and Hall 2016)]. Until recently, angiotensin II receptor antagonists (ARAs) were thought to have effects very similar to those of ACEis (Yusuf et al. 2003; Odagiri et al. 2014). More recent meta-analyses suggest that ACEis, but not ARAs, reduce myocardial infarction and all-cause mortality in patients with hypertension (van Vark et al. 2012; Strauss and Hall 2016). The reasons for these differences are unclear at present (te Riet et al. 2015).

There are several reports that combined statin and ACEi treatment in humans additively improves some mortality and other health-related outcomes (Chae et al. 2011; Zoja et al. 2010; Abdel-Zaher et al. 2012; Faglia et al. 2014). In mice, there are several reports that ACEi treatment increases lifespan (Ferder et al. 1993; Basso et al. 2007; Santos et al. 2009). Others reports indicate ACEi or statin monotherapy have no effect on lifespan (Miller et al. 2011; Harrison et al. 2009). None of these studies report food consumption, body weight, or end-of-life pathologies. In addition, we found no studies reporting the effects of combined statin and ACEi treatment on animal lifespan. For these reasons, we investigated the effects of statin, ACEi, and an ARA monotherapy, and statin and ACEi combination therapy on the lifespan of robust, F1 male mice. The effects of these treatments on food consumption, body weight, and mortality-related pathologies were investigated as well.

## Results

### Lifespan studies

**Fig. 1:**
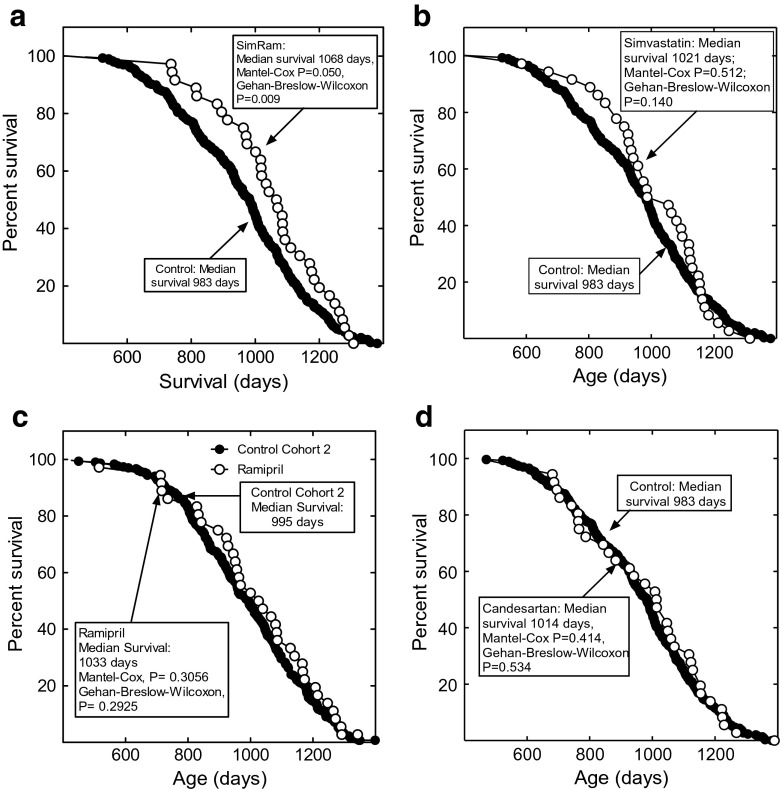
Effects of simvastatin, ramipril, and candesartan monotherapy and SimRam treatment on the lifespan of mice. The effects of SimRam therapy are shown in **a** (*open circles*), while the effects of simvastatin monotherapy are in **b** (*open circles*). The effects of ramipril monotherapy are shown in **c** (*open circles*). The lifespan of candesartan treated mice (*open circles*) is shown in **d** (*open circles*). The control group is shown in all panels as *filled circles*. Feeding of the drugs began at 365 days of age

Male, C3B6F1 mice were treated with simvastatin and ramipril individually and in combination, and with candesartan (Table 1). The rationale for the dosages used is presented in the Discussion. The mouse cohorts and numbers of mice in each group are shown in Table 2. The mice in the control and treatment groups were fed the same number of calories, and food consumption was monitored daily. Combined treatment with simvastatin and ramipril together (SimRam) significantly extended the median and mean lifespan of the mice by approximately 9 %, from 983 to 1068 days (Mantel-Cox *P* = 0.050, Gehan-Breslow-Wilcoxon *P* = 0.009; Fig. 1a). The Mantel-Cox test gives equal weight to all time points, while the Gehan-Breslow-Wilcoxon test gives more weight to deaths at early time points. Monotherapy with simvastatin or ramipril did not significantly change lifespan (Fig. 1b, c). Likewise, the ARA, candesartan, did not alter lifespan (Fig. 1d). Thus, suppression of AT1R signaling is not sufficient to induce the lifespan increase seen in the SimRam-treated mice. Mice fed food containing simvastatin and candesartan together ate so little of the food, even when it contained bacon flavoring, that the studies had to be discontinued for humane reasons.

**Table 1:** Treatment groups and dosages used

Treatment	Concentration in food (mg drug/kg diet)^a^	Approximate dose (mg drug/kg bw/d)^b^
Simvastatin	188	20
Ramipril and simvastatin (SimRam)	47 and 188	5.0 and 20
Ramipril	47	5.0
Candesartan	9.42	1.0

^a^The rationale for the dosages used are in Discussion

^b^Calculated assuming a 39-g mouse, which is the approximate mean body mass throughout the treatment period (Fig. 3a, b)

**Table 2:** Mice cohorts used

Cohort number	Control mouse number	Mouse number per treatment group	Treatment groups
1	297	36	Simvastatin, SimRam, candesartan
2	304	36	Ramipril

**Fig. 2:**
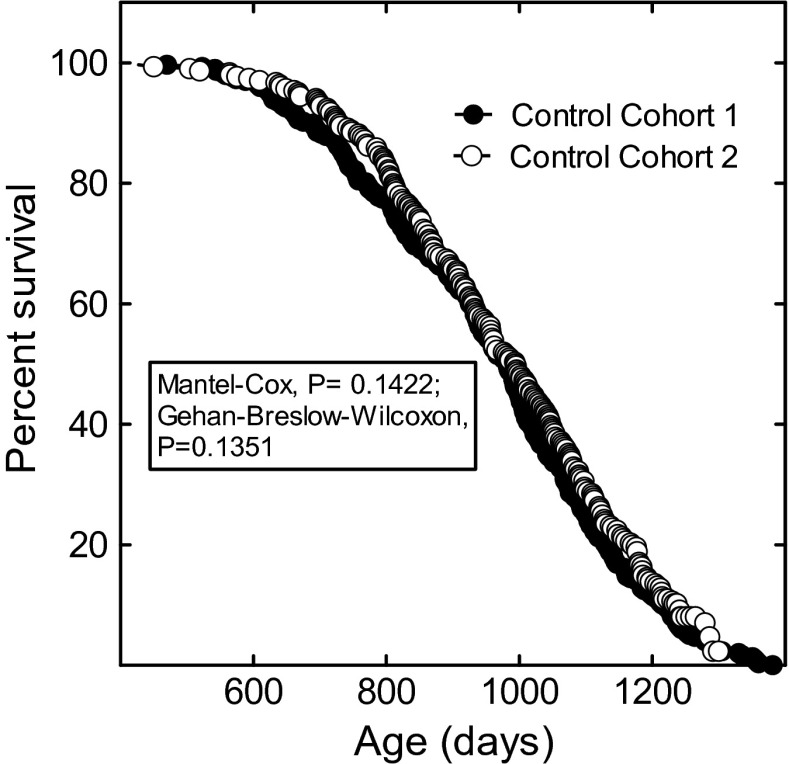
The lifespan of the cohort 1 control mice (*closed circles*) is plotted with the lifespan of the cohort 2 control mice (*open circles*)

The studies reported here are from two lifespan studies, we term cohorts 1 and 2, each composed of 2400 mice. The cohorts 1 and 2 control groups were made up of 297 and 304 mice, respectively (Table 2). All the treatment groups were composed of 36 mice. This unbalanced statistical design allowed the efficient evaluation of a larger number of treatments than would be possible using a balanced design (Jeske et al. 2014). The close equivalency of the two studies is illustrated in Fig. 2, where the survival of the control groups is plotted as a function of age. The median survival of the two cohorts was statistically indistinguishable, 983 and 995 days, for cohort 1 and cohort 2, respectively. The large sample sizes provide a high level of statistical power.

### Drug effects on food intake and body weight

**Fig. 3:**
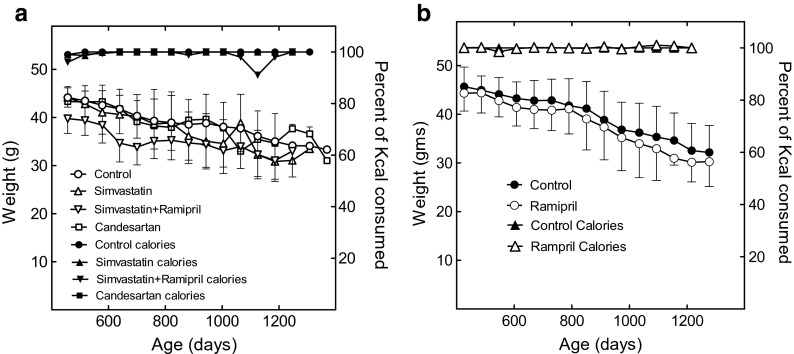
Body weights and food consumption of the mice shown in Fig. 1a, b, d. Shown are the body weights of the control (*open circles*), simvastatin monotherapy (*upward pointing open triangles*), SimRam (*downward pointing open triangles*), and candesartan (*open squares*)-treated mice. Caloric consumption with respect to the number of calories fed is shown for control (*filled circles*), simvastatin (*upward pointing filled triangles*), SimRam (*downward pointing filled triangles*), and candesartan-treated mice (*filled squares*). The statistical significance of these data versus the weights of the control group is shown in Table 3; and discussion in Results. Shown in **b** are the body weights of the control (*closed circles*) and ramipril monotherapy (*open circles*)-treated mice (Fig. 1c). Also shown is caloric consumption for the control (*closed upward pointing triangles*) and ramipril-treated (*open upward pointing triangles*) mice. The ramipril-treated mice were not significantly different in weights or food consumption from the control mice at any time (Table 4; and discussion in Results)

All mice were fed daily, and the groups consumed the same number of calories (Fig. 3a, b). The drugs were cold packed in the AIN-93M diet and fed in measured amounts beginning at 12 months of age. Uneaten food was noted daily, and the next day’s aliquot adjusted to maintain the daily allotment of calories. Food consumption was essentially unchanged throughout the study, indicating that the treatments did not produce “voluntary” caloric restriction (CR).

As observed previously, the mean weight estimates became increasingly unstable as days on diet increased, due to a declining number of survivors (Spindler et al. 2013a; Spindler et al. 2013b). A transient reduction in food consumption by the SimRam group at 1125 days (3 years) of age was related to late-life pathology in 2 of the 12 mice remaining at that time (Fig. 3a). When these mice died, the anomaly resolved. The anomaly in food consumption did not have an effect on mean body mass because body weights were measured before and after the event.

Despite eating the same number of calories, the SimRam-treated mice (SimRam mice) weighed significantly less than the controls, according to a BIC model selection criteria, a likelihood ratio test using a level of significance of 0.01, and an AIC selection criteria (Fig. 3a; Table 3). Ramipril had no effect on body weight (Fig. 3b; Table 4). Together, these data are consistent with the idea that simvastatin disrupts energy homeostasis in mice (see Discussion).

**Table 3:** Summary of the statistical analysis of mouse group weights (Fig. 3a) using BIC model selection removing each diet individually

Diet	*df*	AIC^a^	BIC	*Χ*	Chi *df*	Pr(${\chi}_{df}^2$ > *X*)
Control	16	21,977	22,079			
Simvastatin	14	21,975	22,065	2.3197	2	0.3135
SimRam	14	**22,005**	**22,095**	32.525	2	8.656e-08
Candesartan	14	21,978	22,068	5.5716	2	0.06168

*df* degrees of freedom, *AIC* Akaike’s Information Criterion, *BIC* Bayesian Information Criterion, *Chi df* chi-squared degrees of freedom

^a^Values indicative of significance are shown in bold for convenience

**Table 4:** Summary of the statistical analysis of mouse group weights in (Fig. 3b) using BIC model selection

Diet	*df*	AIC	BIC	*Χ*	Chi *df*	Pr(${\chi}_{df}^2$ > *X*)
Control	12	18,615	18,690			
Ramipril	10	18,613	18,675	1.7653	2	0.4137

*df* degrees of freedom, *AIC* Akaike’s Information Criterion, *BIC* Bayesian Information Criterion, *Chi df* chi-squared degrees of freedom

### Mortality-related pathologies

The necropsy results are summarized in Tables 5 through 8. Few significant differences were found in the pathologies of the control and treatment groups (Tables 5 and 6). There was a significant increase in benign lung tumors in the simvastatin and combined statin treatment groups, and an increase in hemorrhagic diathesis in the SimRam and combined statin treatment groups (Table 5). No differences were found in the mean mass of the liver tumors or total mass of liver tumors per mouse between the control and treated groups (Tables 7 and 8). These differences from control do not appear to have had an impact on survival. Neither the increase in benign lung tumors nor that in hemorrhagic diathesis was consistently associated with altered lifespan.

**Table 5:** Necropsy results for the mice in the longevity studies shown in Fig. 1a, b, d

Organ	Pathology	Dietary treatment													
		Control (*n* = 72)^a^		Simvastatin (*n* = 35)			SimRam (*n* = 36)			Candesartan (*n* = 36)			Combined simvastatin and SimRam^b^ (*n* = 71)		
		No.^c^	(%)^d^	No.	(%)	*P* value^e^	No.	(%)	*P* value	No.	(%)	*P* value	No.	(%)	*P* value
Spleen	Enlarged/tumorous	43	59.7	26	74.3	0.288	14	38.9	0.065	20	55.6	0.685	40	56.3	0.614
Liver	Hemangioma	7	9.7	1	2.9	0.265	3	8.3	1.000	1	2.8	0.265	4	5.6	0.532
	Enlarged/fatty liver	3	4.2	2	5.7	1.000	3	8.3	0.398	6	16.7	0.058	5	7.0	0.494
	Tumor	23	31.9	16	45.7	0.211	13	36.1	0.671	12	33.3	1.000	29	40.8	0.386
Intestine	Tumor	9	12.5	6	17.1	0.566	6	16.7	0.566	1	2.8	0.160	12	16.9	0.488
Lung	Tumor-metastatic	11	15.3	6	17.1	1.000	4	11.1	0.769	1	2.8	0.058	10	14.1	1.000
	Tumor-benign	0	0.0	6	17.1	**0.001**	1	2.8	0.333	2	5.6	0.109	7	9.9	**0.006**
Penis	Necrosed/inflamed	6	8.3	4	11.4	0.728	0	0.0	0.176	0	0.0	0.176	4	5.6	0.745
Seminal vesicle	Enlarged	3	4.2	5	14.3	0.114	2	5.6	1.000	6	16.7	0.058	7	9.9	0.208
Bladder	Distended	11	15.3	4	11.4	0.769	4	11.1	0.769	2	5.6	0.212	8	11.3	0.623
Kidney	Enlarged/tumorous	3	4.2	5	14.3	0.114	2	5.6	1.000	2	5.6	1.000	7	9.9	0.208
Thymus	Enlarged	1	1.4	1	2.9	1.000	2	5.6	0.257	1	2.8	1.000	3	4.2	0.366
Skin/abdominal cavity	Fibroma	4	5.6	2	5.7	1.000	0	0.0	0.299	4	11.1	0.437	2	2.8	0.681
Body cavity	Hemorrhage	11	15.3	10	28.6	0.131	13	36.1	**0.025**	8	22.2	0.425	23	32.4	**0.0190**

^a^Number of necropsied mice in each group. Not all mice in the control group were necropsied. The necropsied control mice were chosen to approximate the ages at death of the SimRam-treated mice

^b^The necropsy results of the simvastatin monotherapy and the SimRam treatment groups in each category were combined and analyzed for differences in occurrence relative to the control group

^c^Number of necropsied mice in each treatment group with the indicated pathologies. One mouse from the simvastatin treatment group was cannibalized and could not be necropsied

^d^The percent of necropsied mice in each group with the indicated pathologies

^e^Fisher’s exact test was utilized to investigate the association between the pathologies and treatments, relative to the controls. This measure of significance used 42 individual tests. The *P* values are two-sided. Values indicative of significance are shown in bold for convenience

**Table 6:** Necropsy results for the ramipril-treated and control mice shown in Fig. 1c

Organ	Pathology	Dietary treatment				
		Control (*n* = 36)^a^		Ramipril (*n* = 36)		
		No.^b^	(%)^c^	No.	(%)	*P* value^d^
Spleen	Enlarged/tumorous	19	70.4	16	59.3	0.569
Liver	Hemangioma	0	0.0	0	0.0	1.000
	Enlarged/fatty liver	1	3.7	1	3.7	1.000
	Tumor	18	66.7	11	40.7	0.101
Intestine	Tumor	2	7.4	1	3.7	1.000
Lung	Tumor	19	70.4	12	44.4	0.098
Penis	Necrosed/inflamed	0	0.0	1	3.7	1.000
Seminal vesicle	Enlarged	2	7.4	1	3.7	1.000
Bladder	Distended	1	3.7	1	3.7	1.000
Kidney	Enlarged/tumorous	2	7.4	1	3.7	1.000
Thymus	Enlarged	5	18.5	7	25.9	0.7445
Skin/abdominal cavity	Fibroma	4	14.8	6	22.2	0.7277
Body cavity	Hemorrhage	7	25.9	9	33.3	0.7664
Thorax only	Hemorrhage	8	29.6	3	11.1	0.1751
Body cavity and thorax	Hemorrhage	15	55.6	12	44.4	0.5867

^a^Number of necropsied mice in each group. All deceased mice were necropsied. The necropsied control mice were chosen to approximate the ages at death of the ramipril-treated mice

^b^The number of necropsied mice with each pathology

^c^The percent of the necropsied mice in each group with the indicated pathologies

^d^Fisher’s exact test was utilized to investigate the association between the pathologies and ramipril treatment. The *P* values are two-sided. No significant differences were found

**Table 7:** Tumor mass for liver tumors reported in Table 5

Liver tumors	Control (*n* = 72)	Simvastatin (*n* = 35)	SimRam (*n* = 36)	Candesartan (*n* = 36)
Mean mass of each tumor ± SEM (g)^a^	1.09 ± 0.18^b^	1.35 ± 0.31 (*P* = 0.684)^c^	1.35 ± 0.37 (*P* = 0.569)	0.68 ± 0.15 (*P* = 0.119)
Tumor mass/number of mice with tumors (g)	1.1	1.7	1.3	0.5

^a^Tumor mass was calculated as (π/6) × l × w × h × 1.0 g, where *l* is the length, *w* is the width, and *h* is the height of each tumor; 1 cm^3^ = 1 g

^b^Mean plus or minus the standard error of the mean

^c^Significance of the difference in median tumor mass calculated using the Mann–Whitney *U* test implemented in Minitab. No significant differences were found

**Table 8:** Tumor mass for liver tumors reported in Table 6

Liver tumors	Control (*n* = 36)	Ramipril (*n* = 36)
Mean mass of the tumors ± SEM (g)^a^	0.88 ± 0.12^b^	0.67 ± 0.19 (*P* = 0.132)^c^
Liver tumor mass/number of mice with tumors	1.2	1.2

^a^Tumor mass was calculated as in Table 7

^b^Mean plus or minus the standard error of the mean

^c^Significance of the difference in median tumor mass calculated using the Mann–Whitney *U* test implemented in Minitab. No significant differences were found

### Blood chemistries

SimRam treatment produced a significant increase in serum glucose concentrations (*P* < 0.001) and a nonsignificant doubling in triglyceride concentrations (Table 9). Ramipril monotherapy produced almost a doubling in serum triglycerides (*P* < 0.01). Ramipril treatment also may have elevated total serum cholesterol, HDL, and LDL, although these changes were only marginally significant. Forty percent CR significantly decreased body weight and serum LDL, while tripling the level of serum triglycerides (*P* = 0.028; Table 9).

**Table 9:** Glucose and lipid profiles of blood serum from control, simvastatin, Sim/Ram, ramipril, and CR-treated mice

	BW	TC	HDL	LDL	TG	NEFA	Glu
	(g)	(mg/dL)	(mg/dL)	(mg/dL)	(mg/dL)	(mEq/L)	(mg/dL)
Control	41.9 ± 3.2^b^	183.1 ± 40.0	175.5 ± 36.9	22.4 ± 8.0	40.8 ± 13.2	0.905 ± 0.157	128.4 ± 13.6
Simvastatin	42.8 ± 2.2	162.9 ± 32.5	162.4 ± 21.7	18.7 ± 7.9	42.5 ± 15.4	1.032 ± 0.231	146.2 ± 40.4
Sim *P* value^a^	0.609	0.404	0.518	0.481	0.853	0.342	0.403
Sim/Ram	39.9 ± 5.0	168.2 ± 25.3	166.9 ± 14.7	20.4 ± 9.7	80.9 ± 36.1	0.901 ± 0.269	**241.4 ± 21.6**
Sim/Ram *P* value	0.489	0.500	0.648	0.726	0.067	0.980	**0.000**
Ramipril	44.16 ± 1.8	231.7 ± 24.1	220.7 ± 23.4	33.9 ± 8.7	**70.7 ± 11.1**	1.051 ± 0.235	130.2 ± 55.4
Ramipril *P* value	0.210	0.060	0.060	0.065	**0.006**	0.292	0.947
40 % CR	**33.9 ± 2.7**	159.3 ± 7.4	141.0 ± 28.7	**9.7 ± 2.6**	**130.8 ± 58.2**	0.729 ± 0.106	122.0 ± 10.6
40 % CR *P* value	**0.004**	0.257	0.143	**0.027**	**0.028**	0.076	0.432

Five male, B6C3F1 mice in each treatment group were 448 days of age at the time they were bled by cardiac puncture. The treated mice received each drug or combination of drugs in their food for 99 days. The 40 % CR group was shifted to 11 kcal/day/mouse of AIN-93 M 20 % Restricted Diet (Diet no. F06298, Bioserv) for 2 weeks, and thereafter to 7.46 kcal/day of AIN-93 M 40 % restricted diet (diet number F05314, Bio-Serv). The stepped reduction in calories reduces stress-induced mortality during the period of initial weight loss. The total time on a CR diet was 99 days. The control mice were fed control diet for the same period of time. Procedures and husbandry were as described for the other mice in this report. Glucose measurements were made using the FreeStyle Lite Blood Glucose Monitoring System (Abbot Diabetes Care, Alameda, CA). Other blood tests were performed by the Comparative Pathology Laboratory, University of California, Davis

*BW* body weight, *CR* calorically restricted, *Glu* glucose, *HDL* high-density lipoprotein, *LDL* calculated low density lipoprotein, *NEFA* nonesterified fatty acid, *TC* total cholesterol, *TG* triglycerides

^a^ *P* values were determined using a two-tailed *t* test. Each *P* value is applicable to the measurements immediately above it

^b^Mean ± standard deviation. Values indicative of significance are shown in bold for convenience

### Endogenous drug levels

Multiple attempts were made to measure the level of simvastatin in the serum of treated mice (Materials and methods). Multiple extraction techniques and liquid chromatography-tandem mass spectrometry (LC-MS/MS) were utilized without success, likely due to the low levels of the drug in serum, and the limited quantities of serum that can be obtained from mice. Similar human studies typically utilize 40 mls of plasma per assay (Zhao et al. 2000). Approximately 3 ml of serum is required to measure serum ramipril concentrations (Lu et al. 2006). Nevertheless, the dosages of the drugs administered to these mice were shown previously to produce well-defined therapeutic endpoints in multiple published mouse studies, including a study from our laboratory (see Drug dosages, in Discussion).

## Discussion

The work presented here shows that treatment with simvastatin and ramipril together extended the lifespan of isocalorically fed, long-lived mice, while monotherapy with either drug alone was ineffective. Candesartan administration was also ineffective at changing lifespan.

### Previous studies

Lifespan extension by SimRam and the decrease in body mass induced by simvastatin in mice are novel results. Our results are consistent with those of others which failed to find a change in lifespan with oral simvastatin treatment (Miller et al. 2011). The decrease in body weight found in our study has not been reported previously, probably because previous studies administered food ad libitum and body weights were not reported.

The lifespan effects of agents which reduced AT1R signaling in rodents have been the subject of conflicting reports (Spindler 2012). Enalapril, an ACEi, has been reported to both increase (Ferder et al. 1993; Basso et al. 2007; Santos et al. 2009) and to have no effect (Harrison et al. 2009) on rodent lifespan. An early study by Ferger et al. found that a greater percentage of mice lived to 24 months of age when enalapril was present in their drinking water (Ferder et al. 1993). However, the treated mice were much heavier than the control mice, and the mean lifespan of the control mice was ∼548 days, which is short relative to that reported by others for this strain (∼664 days) (Kahn 1975). Basso et al. reported that rats treated with enalapril or an AT1R antagonist lived longer than controls (Basso et al. 2007). But, few rats appear to have been involved in this lifespan study, and their actual numbers were not reported. Further, the enalapril-treated rats had lower body weights throughout the study, suggesting that there may have been an unintended CR effect on survival for this group. Santos et al. reported that more enalapril-treated rats lived to 26 months of age than did their chow-fed littermates (Santos et al. 2009). However, both food intake and body weight were significantly reduced by the drug, suggesting the possibility of an unintended CR-related survival benefit. In the studies of Harrison et al., neither body weight nor food intake was reported (Harrison et al. 2009).

### The synergistic effects of simvastatin and ramipril on lifespan

Statins and ACEis are often administered together (DeWilde et al. 2008), and combined treatment additively improves some mortality and other health-related outcomes (Chae et al. 2011; Zoja et al. 2010; Abdel-Zaher et al. 2012; Faglia et al. 2014). For example, diabetic patients with critical limb ischemia had better life expectancies with ACEi and statin therapy combined than when they were treated with either therapy alone (Faglia et al. 2014). Hypertensive, hypercholesterolemic rats and humans undergoing combined statin and ramipril therapy have greater increases in serum nitric oxide and greater reductions in malondialdehyde and high-sensitivity C-reactive protein levels, and reduced systolic and diastolic blood pressure than those treated with either drug alone (Abdel-Zaher et al. 2012). Combined ACEi and statin therapy produces an additive reduction in breast cancer recurrence versus monotherapy with either drug alone (Chae et al. 2011). Likewise, rats with overt diabetic nephropathy who receive combined statin and ACEi therapy have greater reductions in renal proteinuria than with monotherapy using either drug type (Zoja et al. 2010).

A number of mechanisms for the synergistic effects of statins and ACEi on mouse lifespan appear plausible.

#### Energy homeostasis

The decrease in body weight produced by SimRam treatment in the absence of a decrease in food consumption suggests that the drugs produced a change in metabolic energy disposition (Tables 3 and 4). Evidence for this effect can be found in the approximate doubling of blood glucose concentrations and nonsignificant doubling of serum triglycerides (Table 9). These results suggest that SimRam treatment decreased insulin sensitivity. Statins increase the risk of type 2 diabetes and decrease insulin sensitivity and insulin secretion in humans (Sattar et al. 2010; Cederberg et al. 2015). In our mice, ramipril treatment significantly increased serum triglyceride levels, suggesting that this drug also interfered with metabolic control (Table 9). Ramipril treatment has been reported to elevate serum triglycerides in hypertensive, non-insulin-dependent diabetic patients (Schnack et al. 1996). These combined data suggest that SimRam treatment disrupted metabolic control combinatorially in the mice, inducing hyperglycemia and hyperlipidemia.

The association of hyperglycemia and hyperlipidemia with enhanced lifespan seems counterintuitive in that increased insulin sensitivity is most often associated with longer lifespan (Spindler 2010; Bartke et al. 2013). However, rapalogues are robust extenders of mouse lifespan, and they produce hyperglycemia, glucose intolerance, hypertriglyceridemia, and hypercholesterolemia (Miller et al. 2011; Cunningham et al. 2007). In our study, 40 % CR also produced hypertriglyceridemia (Table 9). Like treatment with rapalogues, 40 % percent CR can increase the lifespan of B6C3F1 mice (Spindler et al. 2013b). Thus, SimRam treatment may engage some of the same longevity pathways as rapalogues and CR.

#### Protein isoprenylation

Cholesterol levels were not reduced in the SimRam- or simvastatin-treated mice. Thus, the lifespan effects of SimRam were most likely due to the pleiotropic effects of statins (Table 9). Simvastatin increased the lifespan of *Drosophila* by reducing protein isoprenylation, a non-cholesterol-related effect (Spindler et al. 2012a). Many of the pleiotropic benefits of statins are mediated by reduced isoprenylation of specific small GTP-binding proteins, which results in reduced signaling growth factor receptors (Ludman et al. 2009; Zeichner et al. 2012). The dose of simvastatin used here reduces Ras protein isoprenylation in the B6C3F1 mice used for the present lifespan study (Spindler et al. 2012a). Reduced growth factor signaling is associated with increased lifespan in species as phylogenically diverse as flies and mice (Spindler 2010; Spindler et al. 2012b; Bartke et al. 2013).

#### AT1R signaling

The effects of ramipril on the longevity effects of SimRam may have been the result of reduced AT1R signaling, especially in the vessel wall (te Riet et al. 2015). Decreased AT1R signaling should reduce NAD(P)H oxidase activity in multiple cell types of the vessel wall and elsewhere (Montezano and Touyz 2014; Virdis et al. 2004). This decrease would reduce reactive oxygen species and signaling through mitogen-activated protein kinases, tyrosine kinases, phosphatases, calcium channels, and redox-sensitive transcription factors (Montezano and Touyz 2014; Virdis et al. 2004). Thus, ACEis reduce cell growth and migration, vascular remodeling, and proinflammatory gene expression (te Riet et al. 2015; Zhao et al. 2014; Wu et al. 2014; Muller et al. 2013).

#### Tumors

Upon necropsy of the simvastatin and SimRam groups, we found an increase in the number of benign lung tumors, but no change in other tumor types (Table 5). There is a large population-based study which found that mortality from 13 types of human cancer was reduced by statins (Nielsen et al. 2012). Other studies performed with cultured cells or chemically induced or engrafted human tumor models in mice seem to support this study (Zeichner et al. 2012). However, the behavior of induced, transplanted, and cultured model tumor cells in mice does not necessarily predict the behavior of spontaneous tumors in vivo (Day et al. 2015; Marx 2014). Also, the results of Nielsen et al. have yet to be confirmed in other cohorts. In any case, we found few effects of simvastatin and SimRam on cancer incidence in this study.

### Drug dosages

We utilized drug dosages previously reported to induce well-defined therapeutic endpoints in mice. Simvastatin was administered orally at 20 mg drug/kg bw/day (Table 1). This dose reduces the isoprenylation of specific Ras superfamily members in the liver of the F1 mouse used in the present study and suppresses the growth of K562 chronic myelogenous leukemia cell xenografts in nude mice (Oh et al. 2012; Spindler et al. 2012a). This dose is approximately half that which relieves diabetic complications in db/db mice (Kolavennu et al. 2008). Serum simvastatin levels are too low to be accurately quantified in mice using liquid chromatography tandem mass spectrometry (LC-MS/MS) (Spindler et al. 2012a).

Ramipril was administered at 5 mg/kg bw/day (47 mg drug/kg diet). This dose inhibits multiple measures of atherogenesis in apolipoprotein E-deficient mice (Hayek et al. 2002). Half of this dose reduces pressure overload-induced cardiac hypertrophy and cardiomyocyte and noncardiomyocyte proliferation in male mice (Muller et al. 2013). We did not attempt to measure the serum levels of angiotensin isoforms since *ACE escape* often decouples the blood levels of angiotensin isoforms and aldosterone (which may rise) from the inhibition of their phenotypic activities (which remain inhibited) during long-term ACEi treatment (te Riet et al. 2015). Candesartan was administered at 1 mg/kg bw/day (9.42 mg drug/kg diet). This dose suppresses the growth of human bladder carcinoma cell xenografts in nude mice by 42 % (Kosugi et al. 2009) and increases the survival of a mouse model of inherited dilated cardiomyopathy (Odagiri et al. 2014). The dosages used were chosen for the likelihood that they would be both safe and effective at producing a relevant biological response over the lifespan of a treated mouse.

## Conclusions

Combined simvastatin and ramipril treatment significantly increases the lifespan of B6C3F1 mice. Neither drug alone nor an AT1R antagonist alone significantly altered lifespan. The increase in lifespan in the absence of a change in serum cholesterol or lipoprotein levels recalls studies in *Drosophila* showing that statin monotherapy can increase lifespan by decreasing protein farnesylation and geranylgeranylation (Spindler et al. 2012a). Thus, simvastatin and ramipril combined treatment may extend lifespan by reducing growth factor signaling in mice.

## Materials and methods

### Mouse lifespan and food consumption

Two cohorts of 2400 male B6C3F1 mice were utilized for these studies. The mice were unmated. B6C3F1 mice are a cross between C57BL/6NHsd inbred females and C3H/HeNHsd inbred males. Cohort 1 (Harlan Breeders) included 297 control mice and groups of 36 treated mice. The mice described here were treated with simvastatin, simvastatin and ramipril together, and candesartan at the concentrations shown in Table 1. Cohort 2 (Charles River Laboratories) included a group of 304 control mice and groups of 36 treated mice. One of these groups was treated with ramipril at the concentration shown in Table 1. Other of our reports describe results obtained for other treatment groups in these cohorts (Spindler et al. 2013a, b, 2014a, b, 2015; Martin-Montalvo et al. 2013). After weaning, mice in both cohorts were fed mouse chow (Diet # 5001, Purina Mills, Richmond, IN) ad libitum until random assignment to groups at 12 months of age. Animal husbandry and experimental design were as described in detail elsewhere (Spindler et al. 2013a, 2013b). Briefly, control mice were shifted at 12 months of age to daily feeding with 13.3 kcal/day/mouse of control diet (AIN-93 M, Diet No. F05312; Bio-Serv, Flemington, NJ). Treatment groups (36 mice) were shifted at the same age to daily feeding with 13.3 kcal/day/mouse of control diet mixed with simvastatin, simvastatin and ramipril, ramipril, or candesartan and cold packed into 1 g pellets by Bio-Serv at the dosages shown in Table 1. All mice were fed the same amount of food daily, after the shift. The amount of food males of this strain would eat at 12 months of age was determined by initially feeding the mice ad libitum and reducing feeding weekly until approximately all the food was eaten daily. This was defined as the control amount of food. Mice fed this amount of food are neither fat nor lean and respond robustly to 30 to 40 % CR with extended lifespan [e.g., (Spindler et al. 2013b)]. The food was stored moisture free at 4 °C until used. Food consumption and mouse health were monitored at the time of feeding, and any uneaten food was noted. With the exception noted in the text, all food was eaten daily. The mice were weighed bimonthly. The health of the mice was examined twice daily by laboratory staff, and weekly by a veterinarian. Dead mice were stored at −20 °C until necropsy. This study was approved by the Institutional Animal Care and Use Committee at the University of California, Riverside.

### Statistical analysis of survival, body weight, and food consumption

An unbalanced statistical design was utilized for logistic and economic reasons (Jeske et al. 2014). The statistical power for each group exceeded that required for a Weibull survival analyses with a 75 % probability of detecting a 10 % increase in mean lifespan with a 1 % probability of a false positive (*α* ≤ 0.01) (Jeske et al. 2014). Weibull analyses are more stringent than the Mantel-Cox (Mantel 1966; Peto and Peto 1972; Harrington 2005) or Gehan-Breslow-Wilcoxon tests (Wilcoxon 1945). We utilized Kaplan-Meier survival curves and the Mantel-Cox and Gehan-Breslow-Wilcoxon tests implemented in GraphPad Prism 5.01 to evaluate the significance of the treatments on lifespan. The weight of the mice was determined after an overnight fast every 2 months. The significance of the differences in body weights between the treated and control groups was judged using a linear mixed effects model (Fitzmaurice et al. 2011; McCullagh and Nelder 1989), as described previously (Spindler et al. 2013a). In brief, we modeled the mean response by a set of fixed effects assumed to be shared by mice and a set of random effects that are unique to a particular mouse. Additionally, our model imposed a common intercept since all mice were on the same diet at the time of the first measurement. A Bayesian Information Criterion (BIC) model selection criteria, a likelihood ratio test, and an Akaike’s Information Criterion (AIC) model selection criteria were used to determine significance (Table 3). Food consumption was determined by totaling the amount eaten by each treatment group during a 2-month interval and dividing by number of mice alive during the interval. This value was divided by the amount eaten per mouse by the control group, calculated similarly. The significance of the necropsy results was determined using a Fisher’s exact test.

### LC-MS/MS quantification of simvastatin in serum

Two methods were utilized for sample preparation for LC-MS/MS analysis as described previously (Spindler et al. 2012a). Briefly, for the first method, 200 μL of serum from a simvastatin-treated or untreated mouse was spiked with an internal standard of 1.8 nmol of lovastatin and extracted as described (Zhao et al. 2000). In the second method, sample extraction was performed as described in Lang et al. (1986). The analysis of these extracts by LC-MS/MS was performed as described previously (Spindler et al. 2012a).
